# Using Social Media to Track Geographic Variability in Language About Diabetes: Infodemiology Analysis

**DOI:** 10.2196/14431

**Published:** 2020-02-11

**Authors:** Heather Griffis, David A Asch, H Andrew Schwartz, Lyle Ungar, Alison M Buttenheim, Frances K Barg, Nandita Mitra, Raina M Merchant

**Affiliations:** 1 Children's Hospital of Philadelphia Philadelphia, PA United States; 2 University of Pennsylvania Philadelphia, PA United States; 3 Stony Brook University New York, NY United States

**Keywords:** social media, epidemiology, infodemiology, diabetes, prevalence, twitter

## Abstract

**Background:**

Social media posts about diabetes could reveal patients’ knowledge, attitudes, and beliefs as well as approaches for better targeting of public health messages and care management.

**Objective:**

This study aimed to characterize the language of Twitter users’ posts regarding diabetes and describe the correlation of themes with the county-level prevalence of diabetes.

**Methods:**

A retrospective study of diabetes-related tweets identified from a random sample of approximately 37 billion tweets from the United States from 2009 to 2015 was conducted. We extracted diabetes-specific tweets and used machine learning to identify statistically significant topics of related terms. Topics were combined into themes and compared with the prevalence of diabetes by US counties and further compared with geography (US Census Divisions). Pearson correlation coefficients are reported for each topic and relationship with prevalence.

**Results:**

A total of 239,989 tweets from 121,494 unique users included the term diabetes. The themes emerging from the topics included unhealthy food and drink, treatment, symptoms/diagnoses, risk factors, research, recipes, news, health care, management, fundraising, diet, communication, and supplements/remedies. The theme of unhealthy foods most positively correlated with geographic areas with high prevalence of diabetes (*r*=0.088), whereas tweets related to research most negatively correlated (*r*=−0.162) with disease prevalence. Themes and topics about diabetes differed in overall frequency across the US geographical divisions, with the East South Central and South Atlantic states having a higher frequency of topics referencing unhealthy food (*r* range=0.073-0.146; *P*<.001).

**Conclusions:**

Diabetes-related tweets originating from counties with high prevalence of diabetes have different themes than tweets originating from counties with low prevalence of diabetes. Interventions could be informed from this variation to promote healthy behaviors.

## Introduction

### Background

Diabetes affects 30 million people in the United States, and its prevalence varies by geographic region. A better understanding of the regional differences concerning diabetes could allow for better public health messaging. The colloquial person-to-person communication about diabetes might inform that understanding, but word-of-mouth communication has been hard to measure until social media created the possibility of listening in.

Social media platforms such as Twitter, Facebook, and Instagram have emerged as high-volume, real-time data sources to study and observe communications, including health-related communications, from broad population segments [1-5]. Web-based communities are often far reaching, offering various types of communication including person-to-person communication, information seeking and dissemination, social support, and broadcasting of ideas and opinions. In addition, these communities can have similar location-specific characteristics. The content and characteristics of social media posts are associated with the regional epidemiology of disease [6-8]. For example, Instagram users residing in areas with low access to grocery stores (food deserts) posted about and consumed foods higher in fat and cholesterol compared with users residing in areas with greater access to grocery stores [3]. Thus, a better understanding of how people talk about diabetes via social media could provide insights about how to provide better targeted disease management and treatment.

### Objective

In this study, we sought to characterize language about diabetes on Twitter and examine the correlation between this language and the prevalence of diabetes.

## Methods

### Data Source and Sample

This was a retrospective study of data extracted from Twitter about diabetes. Using natural language processing methodology, we found diabetes-specific terms, grouped them into clusters, and then quantified associations with the prevalence of diabetes. This study was approved by the Institutional Review Board of the University of Pennsylvania.

Tweets are brief status updates (no more than 140 characters during the duration of this study) containing information about emotions, thoughts, behaviors, and other personally salient information. Twitter users are broadly represented across age, geography, and social distributions [9-11]. African Americans, Latinos, and those in urban areas are overrepresented on Twitter relative to the general population [12].

For this study, we examined a random 10.00% (3,700,000/37,000,000) sample of all tweets between July 2009 and February 2015 (37 billion total tweets). We then extracted all tweets in English language with the keyword *diabetes* that originated in the United States, with GPS coordinates or other identifying information sufficient for linking to a US county (such as direct reference to a named county within a state, such as Philadelphia County, Pennsylvania). Approximately 21% of Twitter users provide their location information [5].

### Twitter Topic Generation

We first limited our analysis to diabetes-specific language by finding those words and phrases that had a significant association with posts mentioning diabetes. Specifically, we used a random sample of 25,000 tweets including the word *diabetes* and 25,000 tweets without the word *diabetes*, and out of the 5000 most frequently used words, we kept those that were used significantly more frequently in the diabetes-related messages according to a logistic regression (Benjamini-Hochberg corrected *P*<.05 [13]). This removed nondiabetes-related words such as *the* or *like*. We then grouped diabetes-specific vocabulary in topics (clusters of semantically related words) using Latent Dirichlet Allocation (LDA). LDA is an automated machine learning process by which frequently co-occurring words are organized into topics [14]. Topic usage is quantified on a scale, referred to as *topic probability*, from 0 to 1 (from not used at all to exclusively used), which corresponds to the percentage of words from the given topic.

Two research assistants then independently reviewed 100 topics and categorized them into common themes based on the language within the topics. Any deviations between the research assistants were discussed among the research team members to reach consensus.

### Relation of Diabetes Topics and Prevalence

To determine how topics on diabetes relate to diabetes prevalence, topic probabilities were individually correlated with age-adjusted county diabetes rates from the Centers for Disease Control and Prevention at the county level for 2012 [15]. In addition, topics were regressed against the 9 US Census Divisions using logistic regression controlling for language of the division.

*P* values were corrected for multiple testing using the Benjamini-Hochberg procedure. Pearson correlation coefficients are reported for topics, with *P*<.01 indicating significance.

All statistical analyses were performed with the Differential Language Analysis Toolkit version 1.1 [16] and Python 2.7.10 (Python Software Foundation).

## Results

From approximately 37 billion tweets, 1.8 billion included sufficient location information to map to US counties. Of those, 1.6 billion were in English, of which 239,989 tweets (0.15%) included the term *diabetes*, representing 121,494 unique users.

Topics categorized into themes are displayed in Table 1. Each row of words represents 1 topic within the theme. Examples of topics that correlated with diabetes-related tweets included unhealthy food and drink-themed topics [(*cupcakes*, *whipped*, *Haribo*, and *sundae*) and (*chocolate*, *Cinnabons*, *meats*, and *soda*)] as well as a risk factors theme (*body mass index*, *waist*, *drugs*, *alcoholic*, and *obese)* and a fundraising theme (*walk*, *charities*, *supporting*, *donation*, and *November).*

Twitter users from regions with high prevalence of diabetes were more likely to tweet about unhealthy foods (*candy bar*, *cookies*, and *Twinkies*; *r*=0.088; *P*=.002), whereas twitter users from areas with low prevalence of diabetes were more likely to tweet about research (*clinical*, *published*, and *enrolling*; *r*=0.162; *P*<.001)*.*

**Table 1:** Topics of diabetes-related terms with relevant words within topics, categorized into themes.

Theme	Words within topics
Unhealthy food/drink	Cupcakes, whipped, Haribo, and sundae Fattening, processed, and meats Cinnabons, crispy, and sugar high Kool-aid and lemonade Candy, cookies, and bars Sugar-sweetened, Kentucky Fried Chicken, soda, and Pepsi
Treatment	Exercise, diet, healthy, prevention, and managing Medicine, treatment, symptoms, alternative, natural, and remedies Pancreas, system, physical, and activity Insulin, injections, and sensitivity
Symptoms/diagnoses	Overwhelmed, tiredness, and urination Disease, excess, heart, and hereditary Auto-immune, degenerative, Alzheimer, Crohns, and hyperlipidemia Pregnancy, pre-eclampsia, gestation, and pre-existing Charcot, gangrene, fungal, limbs, and ulcers Unconscious, lightheaded, cramping, and sweating
Risk factors	Obesity, cardiovascular, and dysfunction Obese, antipsychotics, adolescents, and teens Alcoholic, drink, and rum Drugs, statins, women, waist, and body mass index
Research	Mayoclinic.com, lifestyles, and interventions Immunology, antigen, and enrolls Variants, explanation, methylation, and blood
Recipes	Eggplant and recipe Cookbook, ultratasty, health, and recipes Solution, health, and recipe book
News	HealthDay, Yahoo, health news, share, and boost CDC^a^, Americans, worldwide, cases, and percent Rates, CDC rising, and death Syndrome, metabolic, and diagnosis
Health care	Bloodwork, source book, and Dr’s Payer, insurance, professionals, and telemedicine
Management	Glucose, management, monitoring, complications Nurse, pharmacy, education, clinic, patient system
Fundraising	Juvenile, sponsor, walk, annual, research, and donating Walk, step, cure, register, supporting, and donation Charities and revamping Awareness, November, month, national, and advocate
Diet	Mediterranean, diet, reverse, low-carb, high-fat, and paleo Healthy, protein, carbs, meal, and stabilize Plates, lose, eating, weight, and mindful
Communication	Blog, archive, post, and published Community, topic, advocate, and educators Support, group, education, self-management, and wellness
Supplements/remedies	Minerals, raspberries, anti-inflammatory, and chromium Herbs, natural, and alternative care Multivitamin, probiotics, and selenium

^a^CDC: Centers for Disease Control and Prevention.

Themes and topics about diabetes differed in relation to overall prevalence of diabetes across US geographic divisions. Areas with high prevalence of diabetes, such as the East South Central and South Atlantic divisions, also had topics referencing unhealthy food (standardized beta range=0.073-0.146). However, research and exercise were most highly correlated with diabetes prevalence in the Northeast (standardized beta for research and exercise was .107 and .142, respectively).

## Discussion

### Principal Findings

This study reveals that (1) there is variation in what people post on Twitter about diabetes and (2) topics vary by county-level prevalence of diabetes. Unhealthy food–related topics were positively associated with high prevalence of diabetes; conversely, topics about research were negatively correlated with the prevalence of diabetes. The causal directions of these associations, if any, are unclear, but the results suggest opportunities to target online health messages relative to the prevalence of the disease.

This growing body of research utilizing social media platforms to explore public health topics may be helpful for targeting specific patient populations for public health messaging via appropriate language and message content. The ability to relate to different patient populations based on language can better align public health professionals and patients [17,18]. Subpopulations of patients, based on geography, disease severity, or other factors, may use different synonyms or metaphors for symptoms not known to the general public or health professionals. Local health care organizations and professionals could, for example, utilize language common to a particular geographic area with high prevalence of diabetes to target healthy messaging on social media and print media. These organizations may also utilize healthy messaging from other areas with low prevalence of diabetes to influence health behaviors. Large national organizations may also utilize regional differences in content and language to better personalize and position tweets within particular geographic contexts [19].

Content may also be enhanced by tweet modifiers (eg, hashtags and emotion) shown to impact dissemination of cardiovascular health–related Twitter posts [7]. Mining social media to find these nuances within a population posting about diabetes would be useful for outreach and message targeting. Furthermore, learning how different message types (ie, shocking or humorous) are related to gaining knowledge of serious health effects for particular health behaviors is crucial to influence behavior change [2].

### Strengths and Limitations

This study has several limitations. Twitter users are not nationally representative, and tweets are not a direct proxy for all person-to-person communication. Tweets are short, and content is presumably what users are eager to share broadly (vs what they may be focused on privately). Nevertheless, tweets offer a window into public discourse about diabetes. This study also has strengths: it starts from an enormous sample of tweets, systematically addresses their content via machine learning techniques, and associates that content with disease prevalence. In doing so, it advances our understanding of public perceptions of diabetes.

### Conclusions

This study demonstrates that the language used to discuss diseases is variable and complex. Systematic assessment of social media about posts on diabetes could suggest targets for promoting healthy lifestyles and behaviors.

## Abbreviations

LDA: Latent Dirichlet Allocation

## References

[1] Moorhead SA, Hazlett DE, Harrison L, Carroll JK, Irwin A, Hoving C (2013). A new dimension of health care: systematic review of the uses, benefits, and limitations of social media for health communication. J Med Internet Res.

[2] Gough A, Hunter RF, Ajao O, Jurek A, McKeown G, Hong J, Barrett E, Ferguson M, McElwee G, McCarthy M, Kee F (2017). Tweet for behavior change: using social media for the dissemination of public health messages. JMIR Public Health Surveill.

[3] Choudhury D, Sharma S, Kiciman E (2016). Characterizing Dietary Choices, Nutrition, and Language in Food Deserts via Social Media. Proceedings of the 19th ACM Conference on Computer-Supported Cooperative Work & Social Computing.

[4] Ferrara E, Yang Z (2015). Measuring emotional contagion in social media. PLoS One.

[5] Schwartz HA, Kern ML, Dziurzynski L, Lucas RE, Agrawal M, Park GJ, Lakshmikanth SK, Sneha J, Seligman ME, Ungar L (2013). Characterizing Geographic Variation in Well-Being Using Tweets. Proceedings of the Seventh International AAAI Conference on Weblogs and Social Media.

[6] Sinnenberg L, DiSilvestro CL, Mancheno C, Dailey K, Tufts C, Buttenheim AM, Barg F, Ungar L, Schwartz H, Brown D, Asch DA, Merchant RM (2016). Twitter as a potential data source for cardiovascular disease research. JAMA Cardiol.

[7] Eichstaedt JC, Schwartz HA, Kern ML, Park G, Labarthe DR, Merchant RM, Jha S, Agrawal M, Dziurzynski LA, Sap M, Weeg C, Larson EE, Ungar LH, Seligman ME (2015). Psychological language on Twitter predicts county-level heart disease mortality. Psychol Sci.

[8] Weeg C, Schwartz HA, Hill S, Merchant RM, Arango C, Ungar L (2015). Using Twitter to measure public discussion of diseases: a case study. JMIR Public Health Surveill.

[9] (2012). Nielsen.

[10] Duggan M, Brenner J (2013). Pew Research Center.

[11] Epstein JO, Smith N, Xing EP, O'Connor B (2010). A Latent Variable Model for Geographic Lexical Variation. Proceedings of the 2010 Conference on Empirical Methods in Natural Language Processing.

[12] Mislove AL, Ahn Y, Onnela J, Rosenquist J (2011). Understanding the Demographics of Twitter Users. Proceedings of the Fifth International Conference on Weblogs and Social Media.

[13] Benjamini Y, Hochberg Y (1995). Controlling the false discovery rate: a practical and powerful approach to multiple testing. J R Stat Soc Series B Stat Methodol.

[14] Blei DM, Ng AY, Jordan M (2003). Latent dirichlet allocation. Journal of Machine Learning Research.

[15] (2015). Crude and Age-Adjusted Rates of Diagnosed Diabetes per 100 Civilian, Non-Institutionalized Adult Population, United States, 1980-2014.

[16] Schwartz A, Giorgi S, Sap M, Crutchley P, Ungar L, Eichstaedt J (2017). DLATK: Differential Language Analysis ToolKit. Proceedings of the 2017 Conference on Empirical Methods in Natural Language Processing.

[17] Karami A, Dahl AA, Turner-McGrievy G, Kharrazi H, Shaw G (2018). Characterizing diabetes, diet, exercise, and obesity comments on Twitter. Int J Inf Manag.

[18] Semino E, Demjén Z, Demmen J, Koller V, Payne S, Hardie A, Rayson P (2017). The online use of Violence and Journey metaphors by patients with cancer, as compared with health professionals: a mixed methods study. BMJ Support Palliat Care.

[19] Park H, Reber BH, Chon M (2016). Tweeting as health communication: health organizations' use of Twitter for health promotion and public engagement. J Health Commun.

